# P01.53. Spheroid formation and axonal severing in adult neurons during oxidative stress: role of calcium

**DOI:** 10.1186/1472-6882-12-S1-P53

**Published:** 2012-06-12

**Authors:** A Barsukova-Bell, M Forte, D Bourdette

**Affiliations:** 1 Oregon Health & Science University, Department of Neurology, Portland, USA; 2 Vollum Institute, Oregon Health & Science University, Portland, USA; 3 Department of Neurology, Oregon Health & Science University, Portland, USA

## Purpose

Axonal severing is critical to the irreversible disability that occurs over the course of multiple sclerosis (MS). Reactive oxygen species (ROS) are implicated in neurodegenerative aspects of MS: axonal spheroid formation, severing, and axoplasmic Ca^2+^ elevation. However, the exact role of Ca^2+^ in spheroid formation remains unclear. The mechanism of action of natural anti-oxidants such as lipoic acid, which provide neuroprotection during oxidative stress in MS model, also remains unclear.

## Methods

Primary cortical neurons from adult mice were subjected to physiologically-relevant levels of H_2_O_2_. Ca^2+^ dynamics and its sources were examined during spheroids formation using real time imaging, ratiometric Ca^2+^ indicators and immunocytochemistry.

## Results

Exposure to ROS led to a 3.5 fold increase in axoplasmic Ca^2+^ by 30 min. Onset of axonal spheroid formation began at 15 min when Ca^2+^ increase was 2.2 fold. Axonal severing occurred at sites of spheroids around 90-120 min. Analysis of small axonal segments revealed an uneven distribution of Ca^2+^ during exposure to H_2_O_2_. Micrometers apart, focal Ca^2+^ increases in small axonal domains ranged from 2.8 to 4.4 fold. Domains with a 3.8 to 4.4-fold increase correlated with the sites of spheroids, suggesting high focal extracellular Ca^2+^ influx at these sites. Several treatments significantly attenuated Ca^2+^ increase and completely abolished spheroid formation under ROS: removal of extracellular Ca^2+^; N-type Ca^2+^ channel blocker omega-conotoxin GVIA; L-type Ca^2+^ channel blocker amlodipine; and reverse Na+/ Ca^2+^ exchanger (NCX1) blocker KB-R7943. Aggregation of reverse NCX1 and N-type voltage-gated Ca^2+^ channel was detected at spheroids.

## Conclusion

Our results reveal a correlation between focal axoplasmic Ca^2+^ and spheroid formation and suggest that focal aggregation of the reverse NCX1 and N-type Ca^2+^ channel plays central role in high focal Ca^2+^ increase during oxidative stress. These findings provide a basis for investigating the neuroprotective mechanism of the natural anti-oxidant lipoic acid during oxidative stress.

